# Acute Progressive Pediatric Post-Traumatic Kyphotic Deformity

**DOI:** 10.3390/children10060932

**Published:** 2023-05-25

**Authors:** Petr Vachata, Jan Lodin, Martin Bolcha, Štepánka Brušáková, Martin Sameš

**Affiliations:** 1 Department of Neurosurgery, J. E. Purkyně University, Masaryk Hospital, 401 13 Ústí nad Labem, Czech Republic; 2 Department of Neurosurgery, University Hospital in Pilsen, The Faculty of Medicine in Pilsen, Charles University in Prague, 323 00 Pilsen, Czech Republic; 3 Department of Neurology, Masaryk Hospital, 401 13 Ústí nad Labem, Czech Republic

**Keywords:** cervical spine, kyphosis, chin-on-chest deformity, pediatric, trauma, deformity

## Abstract

Cervical kyphosis is a rare entity with challenging management due to the limitations of pediatric age, along with a growing spine. The pathogenesis is made up of a large group of congenital, syndromic and acquired deformities after posterior element deterioration or as a result of previous trauma or surgery. In rare progressive cases, kyphotic deformities may result in severe “chin-on-chest” deformities with severe limitations. The pathogenesis of progression to severe kyphotic deformity after minor hyperflexion trauma is not clear without an obvious MR pathology; it is most likely multifactorial. The authors present the case of a six-month progression of a pediatric cervical kyphotic deformity caused by a cervical spine hyperflexion injury, and an MR evaluation without the pathology of disc or major ligaments. Surgical therapy with a posterior fixation and fusion, together with the preservation of the anterior growing zones of the cervical spine, are potentially beneficial strategies to achieve an excellent curve correction and an optimal long-term clinical outcome in this age group.

## 1. Introduction

The neck is naturally curved in a lordosis, with an average major curve between 14° and 35°, depending on age [1,2,3,4]. The kyphotic alignment is characterized by a deterioration of the global cervical curve below −4° [5]. Kyphotic deformities can be congenital, iatrogenic following posterior laminectomy, the result of infectious or oncological affections, post-traumatic, associated with neuromuscular diseases or aseptic inflammation [3,6,7]. The pediatric population is rarely affected. Progression of the kyphotic curve to a degree that the patient’s chin drops to the chest is called a “chin-on-chest” deformity. This specific type of severe deformity creates the appearance of an obvious humpback. In these cases, the cervical spine curve exceeds 50 degrees. The dropped head syndrome (also known as floppy head syndrome or head ptosis) is a typical example of a chin-on-chest deformity characterized by the severe weakness of the extensor neck muscles, usually due to a neuromuscular disease. Dropped head syndrome is typical for older patients, over 60 years [8]. Severe cervical kyphotic deformity in the pediatric and adolescent population is usually associated with various congenital syndromes; in nonsyndromic cases, severe kyphotic deformities are extremely rare [3,7]. Severe kyphotic deformities may lead to visual angle limitations, dysphagia, speaking and breathing impairment, neck pain, and neurological deterioration due to cervical myelopathy [9]. In general, cervical kyphotic deformity may be treated by conservative techniques (physiotherapy, traction, bracing) or by surgery with anterior, posterior, or combined 360° stabilization and permanent fusion [7]. The authors present a unique case of pediatric post-traumatic progressive cervical kyphotic deformity, without obvious abnormalities on plain radiographs or an MRI, successfully treated by limited posterior cervical fixation and fusion with complete restitution of the physiological curve.

## 2. Case Report

**Figure 1:**
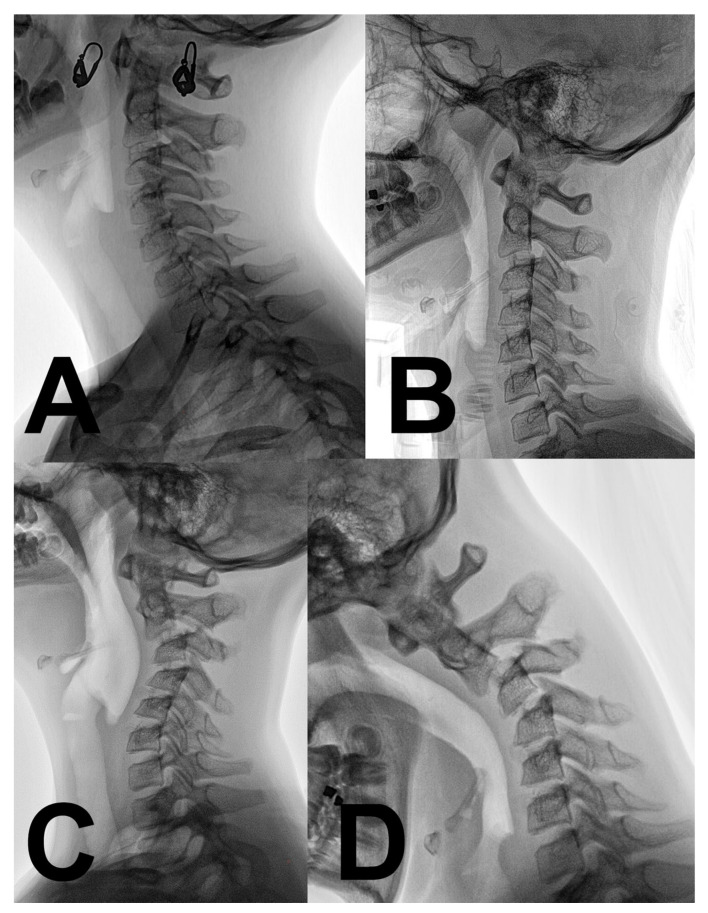
Radiographical evolution of progressive cervical kyphosis after a minor hyperflexion injury. (**A**) Physiological cervical lordosis immediately after a motor vehicle accident. (**B**) Loss of cervical lordosis and C2–3 pseudoluxation one week after trauma (hard collar fixation). (**C**) Cervical kyphosis progression 3 months after trauma. (**D**) Chin-on-chest relief position the deformity 6 months after the trauma, before corrective surgery.

**Figure 2:**
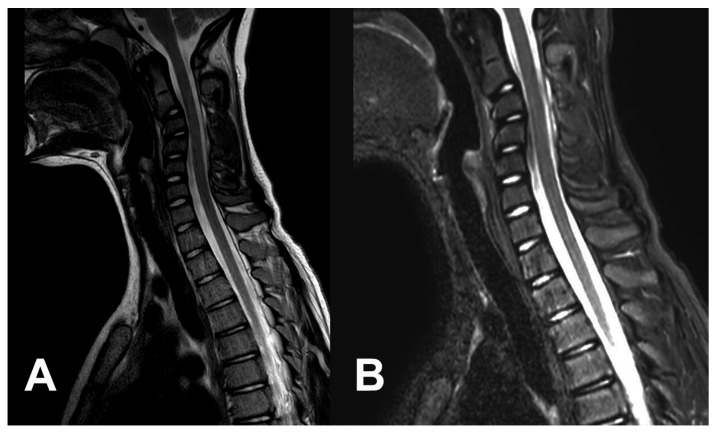
An MR scan performed three weeks after the injury shows that a kyphotic deformity is starting to form, but there are no direct signs of major disco-ligamentous injury. (**A**) T2-weighted sequences. (**B**) STIR-weighted sequences.

An 11-year-old girl (height 161 cm, weight 53 kg, BMI 20) who had no previous medical history of health problems was involved in a car accident. Her parent’s car was crushed by a second car from behind. Both cars were ordinary personal vehicles and the calculated speed difference was 50 km/h. The girl was sitting in the back seat with a seat belt. Hyperextension was limited by standard head support. Hyperflexion was not restricted. There was not any obvious trauma after the accident; the girl only experienced very slight neck pain. Slight neck pain was an isolated sign of trauma during the patient’s evaluation at the local emergency department. Cervical spine radiography was without any signs of trauma (Figure 1). The physiological and neurological status remained unchanged. The movement of the cervical spine was without limitation and without increased neck pain. A hard collar was indicated for one week until the next outpatient department control, and the girl was discharged. One week later, the girl complained of persistent, slight neck pain without any sign of improvement, and was referred to our center. The first radiography control at our trauma center was again without obvious trauma (Figure 1). The lordotic curve was reduced; however, the radiography was performed with a hard collar. No neurological impairment was present; the slight neck pain was the only sign of deterioration. The magnetic resonance (MR) evaluation, including short tau inversion recovery-weighted sequences (STIR) performed two weeks later, revealed an incipient kyphotic deformity of the upper cervical spine with an apex at C3–C4 (Figure 2). There was no sign of bone trauma or intervertebral disc injury. The anterior and posterior longitudinal ligaments and prolonged interspinous ligament were evaluated as intact. The independent second reviewer diagnosed the suspicious lesion of the small interarcuate ligament C34 with a posterior thin deposit of epidural hematoma. Physiotherapy was indicated, but limited due to patient intolerance. The patient repeatedly refused to take off her collar as the pain increased. Throughout the 3-month follow-up, her neck pain increased and a radiography revealed a kyphotic progression of −20° (C2–C7 Cobb) (Figure 1). The patient was still without any signs of radiculopathy or myelopathy. Surgery was recommended to the parents, but they declined the procedure to try a new physiotherapy course at a different facility. During this period, the intolerance of the collar replaced its previous dependency. The neck pain and kyphotic deformity progressed to a chin-on-chest deformity. The patient was admitted to our center for surgery 6 months after initial trauma with severe kyphotic deformity (C2–C7 Cobb) (Figure 1). Active reduction, as well as passive reduction were limited to about 20° by the neck pain. The chin-on-chest position resulted in neck pain relief. No neurological impairment was present. Preoperative electrophysiology (needle electromyography) and laboratory investigations (complete blood count, antinuclear antibody, antineutrophil cytoplasmic antibodies, creatine phosphokinase, antihistidyl transfer RNA synthetase antibody) were without any signs of neuromuscular diseases. Preoperative traction was refused by the parents due to excessive anxiety of their daughter and the risk of potentially increased pain.

**Figure 3:**
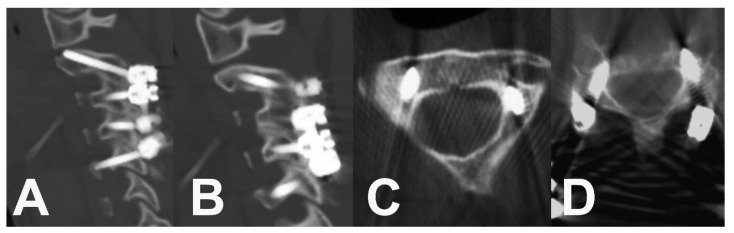
CT scan shows the fusion one year after surgery. (**A**,**B**) Sagittal slices of the fused cervical spine from C2 to C5. (**C**) Axial slice of C2 with transpedicular screw insertion and a high vertebral artery position on the left side. (**D**) Axial slice of the C5 bilateral transpedicular fixation.

**Figure 4:**
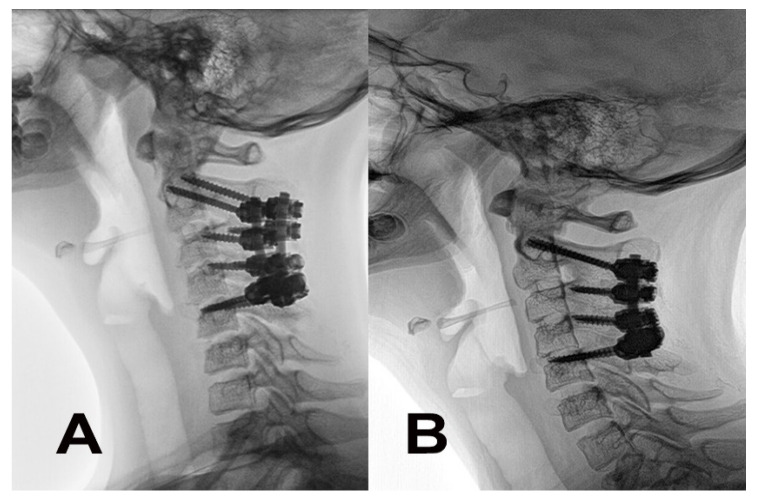
Evolution of the cervical curve after the corrective posterior surgery and fusion. (**A**) Radiography performed immediately after the surgery revealed a correction to a flat, straight curve. (**B**) Restoration of the physiological curve after 3 years.

A posterior approach with an intraoperative reduction and fixation was indicated. Intraoperative traction via a Mayfield head holder under electrophysiological monitoring (MEP, SSEP) reduced the flexible curve to 15°. During the approach, no signs of prior trauma were identified (ligament disruption, disruption of intervertebral joint capsule or segmental instability). A release of the C2–3, C3–4 and C4–5 joints was subsequently performed. Under the control of intraoperative cone-beam CT (O-Arm, Medtronic, Louisville, CO, USA) and navigation (S7, Medtronic, Louisville, CO, USA), pedicle screws to C5 and C2 were successfully inserted, even in the case of a high vertebral artery in C2 on the left side. The pedicles of the C3 and C4 vertebrae were not able to safely accommodate 3.5 mm screws, therefore a lateral mass trajectory (Ellipse, Globus Medical, Audubon, PA, USA) was used. The cartilage of the facet joints and cortical bone in the fusion area were disintegrated by high-speed drilling. Bioactive glass (Bonalive, Bonalive Biomaterials, Turku, Finland) was used to enhance the fusion. The postoperative course was uneventful without any complications. A cervico-thoracic brace (Miami JTO, Össur, Reykjavik, Iceland) was indicated for six weeks. An immediate postoperative X-ray revealed a successful reduction in kyphotic deformity to 0° (C2–C7 Cobb) (Figure 3). The neck pain completely resolved during the first several days after the surgery. Active physiotherapy was initiated six weeks later. CT evaluation one year after fixation revealed a complete posterior fusion from C2 to C5, and the patient completely returned to an active lifestyle without any residual limitations (Figure 4). During her last ambulatory follow-up three years after the correction, the patient was without any problems or limitations. Radiography performed during this last control demonstrated a complete restitution of physiological the cervical lordosis at 16° (C2–C7 Cobb) (Figure 3).

## 3. Discussion

Cervical kyphosis in the pediatric population is rare. The deformity may be congenital or syndromic, as a part of multiple syndromes including Larsen’s syndrome, chondrodysplasia punctata (Conradi’s syndrome), neurofibromatosis, captomelic dysplasia or diastrophic dysplasia [3,7,10,11,12,13,14,15,16]. Acquired insufficiency of the posterior cervical tension band, as in postlaminectomy or post-traumatic cases, is the most common etiology of kyphotic deformity in the pediatric population [17,18,19,20,21,22]. Pal and Shrek evaluated the role of the posterior column (articular processes, facet joints, and ligaments) in cadavers [23]. They demonstrated a dominant 64% of load transmission through the posterior column. Failure of posterior structures is the initiating event, leading to instability and the loss of the physiological lordotic curve. The failure of physiological alignment results in a load axis shift anteriorly to the region of the vertebral bodies. Kyphotic deformity increases compressive loads and increases stress on vertebral endplates. Repeated compressive loading of the head results in wedge rebuilding of the vertebral bodies, together with strain on the posterior cervical muscles, causing neck pain and fatigue. These factors produce kyphotic progression in the growing pediatric spine. The vicious circle should be disconnected in time to avoid draping and stretching of the spinal cord over the kyphotic apex with subsequent myelopathy [7,9].

In our case, traumatic hyperflexion of the cervical spine during the motor vehicle accident was the initiating pathological factor. Despite the absence of any osseous trauma, an intact physiological curve of the cervical spine and minimal clinical symptoms, the curvature progressively deteriorated to a chin-on-chest deformity throughout several months. The first radiography control a week later revealed a slight deterioration of the cervical curve. The loss of lordosis corresponded to collar fixation [24,25]. The benign C2–3 pseudosubluxation has also been described [24,25,26]. The subsequent MR and intraoperative evaluation excluded any major trauma to the posterior ligamentous complex and intervertebral discs. The initial factor was very likely only minimal ligamentous and muscular trauma. On the other hand, neck pain was probably the most important factor in the rapid progression of kyphosis. In our case, the so-called idiopathic acute progressive adolescent cervical kyphosis corresponds most closely with the time course of deterioration. Acute adolescent idiopathic kyphosis is by definition a cervical deformity without an identifiable cause, with a high risk of progression to a severe degree of kyphosis, including chin-on-chest deformity [6,9,27,28]. Postural habits, neck pain and weakness of the extensor muscles may be the potential underlying factors in these cases [6,29]. Psychological factors may also contribute to the progressive nature of kyphosis. The same factors presumably played a similar role in our case after the initial trauma. The large number of studies published in Asia may suggest a possible genetic predisposition.

A typical example of chin-on-chest deformity is the dropped head syndrome (DHS) [8,30,31]. This type of severe kyphotic deformity results from the decreased strength of extensor muscles due to different, mostly neuromuscular diseases, such as amyotrophic lateral sclerosis (ALS), Parkinson’s disease, polymyositis, myasthenia gravis, genetic myopathies, motor neuron disease, or post-radiation atrophy [8,30,32]. In cases without a secondary cause, the condition is called the isolated neck extensor myopathy. The incidence of DHS is limited to older adults, usually between 60 and 70 years old. The disease can have a progressive or sudden onset in character [33]. A review published by Drain et al. identified 129 patients with DHS described in 74 studies [8]. Cavagnaro et al. published a systematic review of 54 patients treated by surgery [34]. Cervico-thoracic arthrodesis, usually from C2 to Th3 or Th4, was the most common surgical procedure. Limited fusion without a thoracic spine component resulted in a 71% failure rate. The surgery was performed from a posterior-only approach or as a combined procedure. An anterior approach resulted in dysphagia in 75% [34]. A completely different surgical strategy for this flexible, severe kyphotic deformity was described by Farshad et al. as occipitopexy [35]. This correction was based on a ligamentous fixation of the occiput to the upper thoracic spine without fusion. In our case, the electrophysiological evaluation excluded any neuromuscular diseases preoperatively.

Treatment modalities for cervical kyphosis include conservative and surgical measures. Conservative strategies such as physiotherapy, chiropractic procedures, anti-inflammatory agent applications, steroid injections, bracing, or cervical traction are limited to a low-degree kyphosis without neurological deterioration [28]. The effects of these conservative methods are not sufficiently supported by any data. The cervical collar itself may improve the kyphotic curve, but also deteriorate the physiological lordotic curve. The proper timing of bracing to prevent kyphotic deterioration, but not increase the risk of extensor muscle weakening is not established either. Surgical treatment is usually required in cases where a deformity progressively forms along with neck pain and an increased risk of neurological deterioration [3,7,28]. Preoperative head traction may be beneficial in improving the curve prior to the final surgical release and fixation [36,37]. However, neck pain is a possible limitation to traction, such as in our case. Current surgical strategies for treating pediatric cervical kyphotic deformity are based on several retrospective studies with a limited number of cases [3,7,10,38]. The all-inclusive series is made up of a heterogenous group of kyphotic deformities. The anterior, posterior, or combined 360° approaches may be used to stabilize and permanently fuse the pathological cervical curve. The anterior approach was historically preferred because of its familiarity; however, the results with a higher incidence of cage subsidence, pseudoarthrosis, and failure of curve correction. As such, there has been a preference for posterior approaches with the aid of pedicle screws with higher stability and pullout strength [39,40]. The anterior approach alone or as a part of 360° reconstructions is limited to rigid curves. In severe curves, the combined approach resulted in a lower rate of pseudoarthrosis and a better deformity correction than a single could achieve [40,41]. The posterior approach is the method of choice for flexible curves [38]. The physiological growth and coupled change of alignment of the pediatric cervical spine should be taken into consideration. Continued growth of the cervical spine occurs in two-thirds of pediatric patients after rigid titanium instrumentation and permanent fusion [42]. Goldstein et al. described 83% of persistent vertical growth after a 3-level construct [42]. The destruction of the endplates by anterior grafting combined with posterior instrumentation is identified as a risk factor for the atrophy of vertebral bodies [43]. Our case illustrated the successful combination of physiological growth zone preservation with a posterior correction, fixation, and fusion in a flexible severe kyphotic deformity, leading to an excellent medium-term result.

## 4. Conclusions

Severe kyphotic deformities are extremely rare entities within the pediatric population. The treatment schedule is not supported by strong evidence based on randomized or prospective studies. Only a few limited retrospective studies with high heterogeneity in the etiology and pathogenesis of kyphotic deformity were published. The long-term results are compromised by the physiological growth of the cervical spine in this age group. The case demonstrates a unique progressive kyphotic cervical deformity after whiplash-type trauma without any obvious MR pathology of disc or major ligaments, resulting in a chest-on-chest deformity. A second independent reviewer of the initial MRI scan diagnosed a suspicious lesion of small interarcuate ligament C3/C4 and a small posterior epidural hematoma. After the failure of conservative treatment, posterior fixation and fusion was indicated. Posterior fixation with a growing zone preservation resulted in complete restoration of physiological cervical lordosis.

## Data Availability

Not applicable.
